# Exploring the Changes in Mild Cognitive Impairment Blood-Based Biomarkers after Local Antibiotic Periodontal Treatment in Diabetic Patients: Secondary Analysis of Data from a Randomized Controlled Trial

**DOI:** 10.1055/s-0044-1795115

**Published:** 2024-12-30

**Authors:** Aulia Ramadhani, Azusa Tanaka, Kumiko Minagawa, Sachiko Takehara, Takaho Yamada, Hirohito Sone, Noboru Kaneko, Kaname Nohno, Hiroshi Ogawa

**Affiliations:** 1 Division of Preventive Dentistry, Graduate School of Medical and Dental Sciences, Niigata University, Niigata, Japan; 2 Department of Dental Public Health, Faculty of Dental Medicine, Universitas Airlangga, Surabaya, Indonesia; 3 Niigata Public Health and Sanitation Center, Niigata, Japan; 4 Department of Hematology, Endocrinology and Metabolism, Faculty of Medicine, Niigata University, Niigata, Japan; 5 Department of Oral Health and Welfare, Graduate School of Medical and Dental Sciences, Niigata University, Niigata, Japan

**Keywords:** periodontitis, diabetes, mild cognitive impairment, periodontal treatment, inflammation

## Abstract

**Objectives** This article investigates the changes in blood-based biomarkers associated with mild cognitive impairment (MCI) risk in type 2 diabetic patients following local antibiotic periodontal treatment.

**Materials and Methods** A secondary analysis of data from a 24-week randomized controlled trial was conducted, involving 27 patients with type 2 diabetes mellitus and periodontitis. Participants received periodontal treatment biweekly from baseline until the 6th week of the study. Fourteen patients in intervention group received scaling with local antibiotic adjunct (Periofol 2%). The outcomes were periodontal inflammation score, which was measured using periodontal inflamed surface area, the inflammation markers levels (tumor necrosis factor-α, C-reactive protein, and interleukin [IL]-6), and MCI risk score, which was assessed using protein plasma analysis through blood test. The evaluations were performed at baseline and week 24th in both groups. The changes in periodontal inflammation scores, inflammation parameters, and MCI risk in baseline and week 24th were analyzed.

**Statistical Analysis** The Wilcoxon signed-rank test was used for within-group analysis and the Mann–Whitney *U* test was utilized for between-group analysis.

**Results** Periodontal parameters were improved in both groups ( *p* < 0.05). IL-6, complement C3, and alpha-2-antiplasmin levels were significantly decreased in the intervention group ( *p* < 0.05). In between-group comparisons, there was a significant difference between the control and intervention groups in apolipoprotein A1, apolipoprotein C1, and alpha-1-B glycoprotein levels in week 24th ( *p* < 0.05).

**Conclusion** Even though the periodontal status showed significant improvement after being given local antibiotic periodontal treatment, the changes in MCI risk proteins plasma remained unclear.

## Introduction

Type 2 diabetes mellitus (T2DM) is a chronic metabolic disorder characterized by persistent hyperglycemia caused by resistance to peripheral actions of insulin. 1 Nowadays, diabetes mellitus (DM) is a major public health concern in Japan with 10 million people (12% of population) are suspected of having the disease. 2 It is predominated by T2DM in both adults and children. 3 In addition to its well-known effects on glycemic management and related problems, emerging evidence points to a possible relationship between T2DM and cognitive decline, as well as an elevated risk of mild cognitive impairment (MCI). MCI is the preclinical, transitional stage between healthy aging and dementia, its early detection and risk factor management are crucial for targeted interventions and prevention strategies. 4 A previous study in Japan shows that among the diabetic population, 11.9% of the population was diagnosed with dementia. 5 Although there is debate concerning the link between DM and poor cognitive performance, the evidence for this association is growing. Another previous 7-year longitudinal study on 828 subjects found an increased risk for Alzheimer's disease (AD) in subjects with DM. 6

The possibility of an association between periodontal disease and cognitive impairment in people with T2DM is an exciting topic of research that has attracted interest lately. Periodontitis is an oral disease affecting the periodontium's hard and soft tissue, and it commonly happens as people age. It has been suggested that, particularly in vulnerable groups like those with T2DM, the systemic inflammation linked to periodontal disease may contribute to the onset or progression of cognitive impairment. 7 8 9

Both periodontal inflammation and DM are the potential modifiable risk factors of MCI, moreover, they are also related to each other and have a “two-way relationship.” 10 We hypothesize that reducing the systemic inflammation caused by periodontitis in diabetic patients will reduce the risk of MCI. Emerging studies found protein plasma from blood samples is a potential biomarker for MCI prevention. 11 12 Based on available evidence, this study aims to explore the changes in MCI risk blood-based biomarkers in type 2 diabetic patients after local antibiotic periodontal treatment.

## Materials and Method

### Study Design

This research is a secondary data analysis from a randomized controlled trial comprising a two-group parallel design with a single-blind study, called “Effects of Periodontal Treatment on the Risk of Cerebral Infarction in Patients with Type 2 Diabetes Mellitus” registered under clinical trial registry number UMIN000042458 with the University Hospital Medical Information Network (UMIN) Center and received approval and ethical clearance (number 2020-0126) from the Niigata University Ethical Board. Each participant provided verbal and written informed consent to participate in the study. The study was conducted for a total of 24 weeks for each patient. The time measured for each week might differ, depending on the starting week of the patients. During week 0 (baseline) and week 24th, both groups received medical, periodontal examination, and blood sampling. Periodontal treatments were given in week 0, week 2, week 4, and week 6.

### Study Participants

A total of 30 T2DM patients were recruited from the Department of Endocrinology at Niigata University Hospital from November 2020 to April 2023. Patients who met the following medical and dental criteria (screened by T.Y. and H.O.) were included: hemoglobin A1c (HbA1c) ≥ 6.0%, absence of insulin therapy, maintenance of a stable diabetic treatment regimen for the preceding 2 months, absence of a history of stroke, nonsmoker, and presence of at least 10 teeth with 4 periodontal pockets measuring 4 mm or more in-depth. Patients who met the inclusion criteria were divided into antibiotic periodontal treatment (intervention) or nonantibiotic periodontal treatment (control) groups. Patient assignment was conducted using the block randomization method (assigned by H.O.). Patients will not be included if their HbA1c is less than 6.0%, if there has been a change in diabetes treatment within the past 2 months, or if they have a history of cerebral infarction.

### Periodontal Treatment

**Fig. 1:**
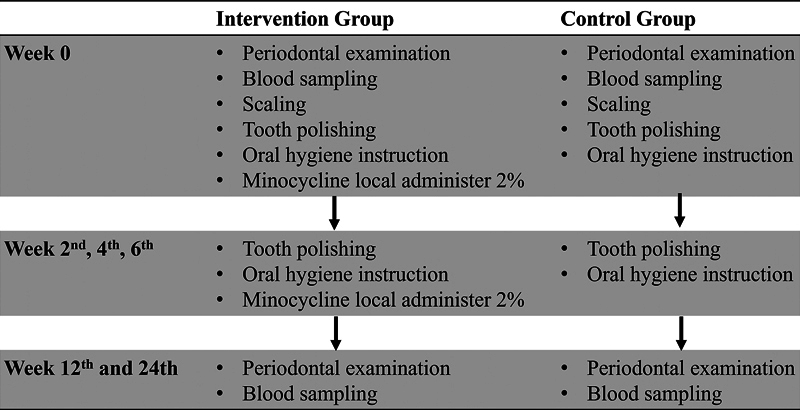
Research intervention protocol.

Periodontal treatments were performed for both the intervention and control groups, with distinct protocols implemented by K.M. and A.T. in the Preventive Dentistry Clinic, Niigata University, Japan. In the intervention group, procedures included scaling, tooth polishing, oral hygiene instruction, and application of 10 mg minocycline antibacterial agent (Periofol dental ointment 2%, Showa Yakuhin, Tokyo, Japan). After scaling, the gel is carefully administered into the periodontal pockets, specifically targeting areas with probing depths of 4 mm or greater. Subsequently, the intervention group received tooth polishing, antibacterial agent application, and oral hygiene instruction every 2 weeks from weeks 2 to 6. Conversely, the control group received oral hygiene instruction tooth polishing every 2 weeks throughout the study period (week 0–6). The intervention protocol is visually represented in Fig. 1 .

### Outcome Variables and Instruments

Multiple variables of general health were assessed in this study to describe its relationship with periodontal disease. According to the aim of the current study, four variables were selected.

1. General health variables: Age, gender (female, male), body mass index (BMI), and HbA1c level.
2. Periodontal status variables: The periodontal examinations were conducted at the Department of Preventive Dentistry, Niigata University Hospital, by a single examiner (H.O.) using mouth mirrors and World Health Organization (WHO) Community Periodontal Index probes (Perio probe WHO, YDM Co., Ltd., Tokyo, Japan) under artificial lighting. The probing pocket depth (PPD), clinical attachment level, and bleeding on probing were conducted at baseline (week 0), week 12, and week 24 at six sites per tooth: buccal, lingual, mesiobuccal, mesiolingual, distobuccal, and distolingual surfaces. Probing was carried out at six points per tooth for all present teeth, applying a force of approximately 20 to 25 g, with measurements rounded to the nearest millimeter. The collected data were utilized to compute the periodontal inflammatory burden using the periodontal inflamed surface area (PISA) method, expressed in square millimeters, employing an Excel spreadsheet designed for PISA scoring. 13
3. Inflammation markers variable: Tumor necrosis factor-α (TNF-α), C-reactive proteins (CRPs), and interleukin-6 (IL-6). These variables were taken from one portion of blood samples (2 × 5 mL) analysis, which was collected from each participant in the Central Blood Collection Room, Niigata University Medical and Dental Hospital. Subsequently, the samples were centrifuged at 3,000 revolutions per minute and promptly stored in a freezer (BML Corporation, Japan).
4. MCI risk variables: The second portion of the samples were designated for the analysis of MCI risk. The proteins involved in the assessment are the amyloid-β sequester proteins (apolipoprotein A1 [ApoA1], complement C3, and transthyretin [TTR]), 11 the nutrition-related proteins (albumin), lipid metabolism protein (apolipoprotein C1 [ApoC1]), innate immunity and inflammation (alpha-1-B-glycoprotein [A1BG]), and coagulation-fibrinolytic protein (alpha-2-macroglobulin [A2M], alpha-2-antiplasmin [A2AP], and hemopexin [HPX]). 12 MCI risk protein assessment was conducted by MCI Plus Company (MCI Screening Test Plus, MCBI Co, Ltd., Japan). The methods for plasma protein analysis followed protocol outlined in Inoue et al. 12 The process includes:

1. Plasma sample preparation and protein digestion.
2. Liquid chromatography-tandem mass spectrometry analysis.
3. Protein identification and quantification.

### Statistical Analysis

Statistical analysis was (performed by A.R., S.T., K.N.) using IBM SPSS version 28 (IBM Corp, New York, United States). Due to the abnormal distribution of the data, nonparametric tests were applied. All results will be presented as median values (interquartile range) unless otherwise stated. Changes in all parameters from baseline to week 24 were compared using the Wilcoxon signed-rank test with a 95% confidence interval (CI) (GraphPad Prism version 10.0.0 for Windows, GraphPad Software, Boston, Massachusetts, United States, www.graphpad.com ). The Mann–Whitney *U* -test was utilized to compare parameters between the intervention and control groups ( *p* < 0.05).

## Results

### Study Population and Baseline Characteristics

**Fig. 2:**
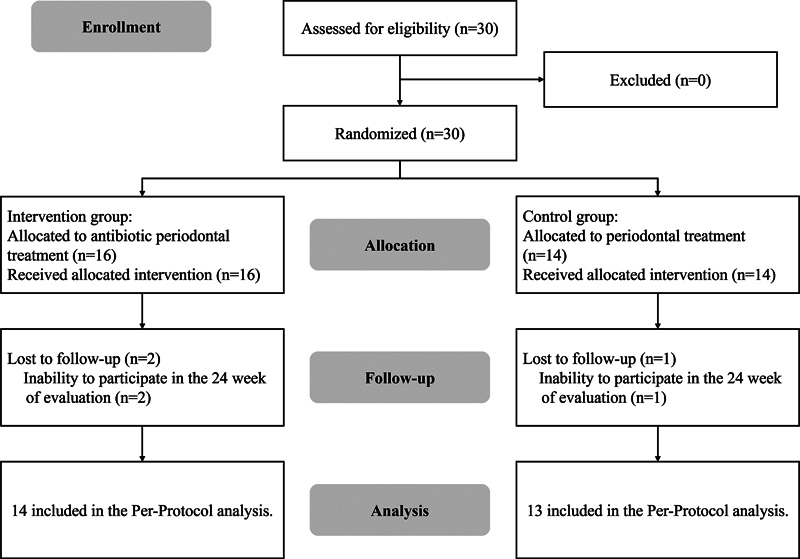
Study population flow.

The results of a total of 30 patients (16 patients assigned to the intervention group and 14 patients assigned to the control group) were analyzed. However, two patients from the intervention group and one from the control group were excluded from the analysis due to their inability to participate in week 24th of their examination ( Fig. 2 ). Table 1 shows the baseline characteristics of the respondents. There was no significant difference in age, BMI, and HbA1c between the control and intervention groups on baseline. Four patients had PISA score of more than 300 in the intervention group.

**Table 1:** Patients baseline characteristics

	Control group ( *n* = 13)	Intervention group ( *n* = 14)
Age	64.9 ± 12.0	65.3 ± 9.6
Gender		
- Female	6 (46.2%)	5 (35.7%)
- Male	7 (53.8%)	9 (64.3%)
BMI (mean ± SD)	24.6 ± 3.9	26.4 ± 3.2
HbA1c (mean ± SD)	7.1 ± 0.6	7.0 ± 0.7
PISA		
- < 300	13 (56.5%)	10 (43.5%)
- > 300	0 (0.0%)	4 (100%)

Abbreviation: BMI, body mass index; HbA1c, hemoglobin A1c; PISA, periodontal inflamed surface area; SD, standard deviation.

Note: Values for normally distributed data present in means ± SD.

### Effect on Periodontal, Systemic, and MCI Risk Parameters

**Fig. 3:**
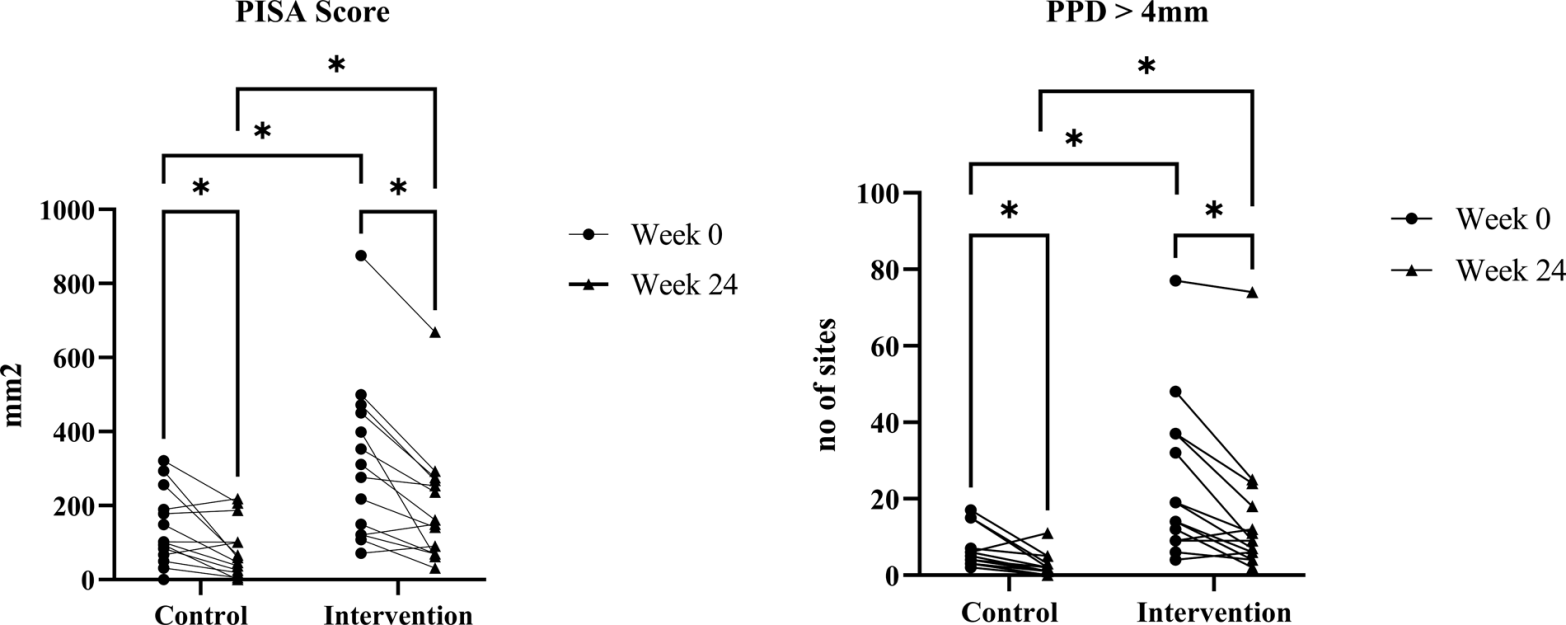
Comparison of values changed in periodontal parameters between baseline and week 24. *Wilcoxon signed-rank test, a significant difference compared with baseline ( *p* < 0.05).

Table 2 presents the periodontal, systemic, and MCI risk parameters in control and intervention from baseline to week 24. The Mann–Whitney *U* -test was used to analyze between-group comparison, and the Wilcoxon signed-rank test was used for within-group comparison over time (95% CI). There was a significant difference between the intervention and control group in between-group comparison ( *p* ^b^ ) of PISA score and number of PPD > 4 mm. Meanwhile, for within-group comparison (compared to baseline data) ( *p* ^a^ ), there was a significant difference in PISA score and PPD > 4 mm between baseline data and week 24 in both groups. In the intervention group, we can see improvements in a number of sites with probing depth of more than 4 mm between baseline, week 12 ( *p* < 0.05), and week 24 ( *p* < 0.01), while in the control group, the significant difference was only shown between baseline and week 24 ( *p* < 0.05) ( Fig. 3 ).

**Table 2:** Periodontal, systemic, and MCI risk parameters in the control and intervention groups over time

Parameters		Control group	*p* a	Intervention group	*p* a	*p* b
PISA (mm ^2^ )	Baseline	102.1 [75.3–222.8]	0.02 *	293.7 [121.1–458.5]	< 0.01 *	0.01 *
	Week 24th	59.6 [22.1–144.1]		155.3 [69.8–269.8]		0.01 *
PPD > 4 mm	Baseline	6.0 [3.5–11.0]	0.01 *	16.5 [9.0–37.0]	< 0.01 *	< 0.01 *
	Week 24th	2.0 [1.0–4.0]		9.0 [5.5–19.5]		< 0.01 *
Systemic parameters						
IL-6 (pg/mL)	Baseline	1.7 [1.0–2.9]	0.70	2.0 [1.2–5.9]	0.02 *	0.30
	Week 24th	1.6 [1.2–3.1]		1.8 [0.7–3.9]		0.92
TNF-α (pg/mL)	Baseline	0.9 [0.6–3.6]	0.06	1.2 [0.7–2.1]	0.17	0.51
	Week 24th	0.8 [0.4–0.9]		0.8 [0.5–1.3]		0.33
CRP (mg/dL)	Baseline	0.0 [0.0–0.1]	0.36	0.0 [0.0–0.5]	0.04 *	0.04 *
	Week 24th	0.0 [0.0–0.0]		0.0 [0.0–0.1]		0.14
MCI risk parameters						
Lipid metabolism proteins						
TTR (mg/dL)	Baseline	20.4 [19.0–23.6]	0.01 *	20.5 [17.9–25.6]	0.34	0.90
	Week 24th	22.6 [20.8–28.6]		22.9 [19.8–28.3]		0.77
ApoA1 (mg/dL)	Baseline	195.0 [152.8–229.9]	0.86	167.7 [155.1–185.8]	0.10	0.28
	Week 24th	189.3 [161.2–203.2]		165.5 [130.2–183.5]		0.02 *
ApoC1 (mg/dL)	Baseline	6.5 [5.1–8.0]	0.50	5.2 [4.5–6.6]	0.06	0.16
	Week 24th	7.2 [5.0–7.5]		4.5 [4.1–6.6]		0.01 *
Inflammation and immunity proteins						
C3 (mg/dL)	Baseline	118.7 [107.5–132.3]	0.67	120.5 [112.2–131.2]	0.02 *	0.46
	Week 24th	122.4 [106.7–133.6]		111.5 [107.5–119.3]		0.22
A1BG (mg/dL)	Baseline	26.3 [20.5–30.0]	0.46	23.8 [19.7–26.0]	0.06	0.19
	Week 24th	27.9 [23.8–33.1]		22.5 [19.1–24.5]		0.01 *
Coagulation proteins						
HPX (mg/dL)	Baseline	104.6 [92.0–121.8]	0.19	112.2 [101.1–128.6]	0.14	0.28
	Week 24th	111.8 [102.0–116.7]		111.4 [95.8–126.3]		0.88
A2AP (mg/dL)	Baseline	6.4 [5.6–7.1]	0.34	6.5 [5.9–7.1]	< 0.01 *	0.67
	Week 24th	5.9 [5.0–6.8]		5.8 [5.0–5.9]		0.39
A2M (mg/dL)	Baseline	232.0 [185.3–338.9]	0.42	252.0 [217.4–296.8]	0.73	0.77
	Week 24th	244.5 [198.7–315.6]		258.7 [214.0–290.8]		0.92
Nutrition proteins						
Albumin (g/dL)	Baseline	5.2 [4.7–5.8]	0.65	5.2 [4.9–5.6]	0.13	0.92
	Week 24th	5.5 [4.9–5.6]		5.1 [4.8–5.2]		0.07

Abbreviations: A1BG, alpha-1-B-glycoprotein; A2AP, alpha-2-antiplasmin; A2M, alpha-2-macroglobulin; ApoA1, apolipoprotein A1; ApoC1, apolipoprotein C1; C3, complement C3; CRP, C-reactive protein; HPX, hemopexin; IL-6, interleukin-6; MCI, mild cognitive impairment; PISA, periodontal inflamed surface area; PPD, periodontal probing depth; TNF-α, tumor necrosis factor-α; TTR, transthyretin.

Note: All values are presented in median [interquartile range].

a *p* : Wilcoxon signed-rank test, significant difference compared with baseline ( *p* < 0.05).

b *p* : Mann–Whitney *U* test, significant difference between control and intervention group ( *p* < 0.05).

* significant *p* -value <0.05.

In systemic inflammation parameters assessment, a significant difference in IL-6 between baseline and week 24 in the intervention group ( *p* = 0.02) was observed. No significant difference was observed in the other variables (HbA1c, TNF-α, and CRP). Diabetic parameters were represented through HbA1c levels. For both groups, the level of HbA1c tends to decrease over time.

**Fig. 4:**
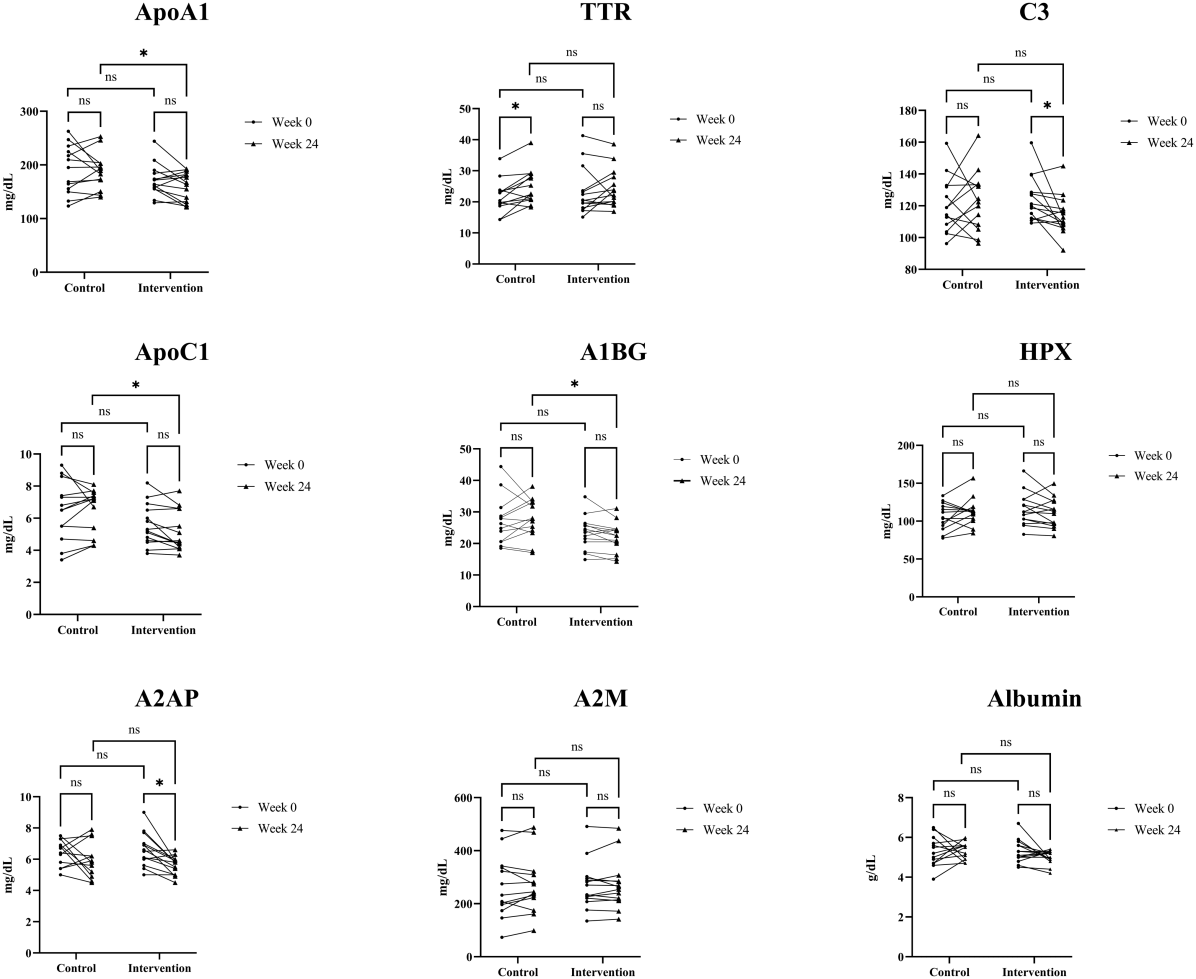
Comparison of values changed in systemic and mild cognitive impairment (MCI) risk parameters over time. *Wilcoxon signed-rank test, a significant difference compared with baseline ( *p* < 0.05); ns, not significant. PISA, periodontal inflamed surface area; IL-6, interleukin-6; ApoA1, apolipoprotein A1; TTR, transthyretin; C3, complement C3.

Based on Table 2 , there was a significant difference in week 24 assessment between the control and intervention group in ApoA1 ( *p* = 0.02). The C3 variables in the intervention group show a significant difference in median value between baseline and week 24 ( *p* < 0.05). Significant changes from baseline to week 24th in MCI risk parameters were observed in the intervention group, such as C3 and A2AP. Meanwhile, there were a significant difference between the control and intervention group in week 24th that can be observed in ApoA1, ApoC1, and A1BG. The changes in MCI risk parameter are shown in Fig. 4 .

## Discussion

DM, periodontitis, and cognitive impairment share common risk factors and have been known to be connected through inflammatory pathways. A study in 2022, conducted by Balasubramaniam, discovered a significant number of diabetic individuals with chronic periodontitis having possible chances of developing cognitive impairment. 14 Another study suggests that the risk of cognitive impairment may increase by high IL-6 levels. 15 This study aimed to explore the effects of antibiotic periodontal treatment on MCI risk through the changes in protein plasma from blood samples assessment in T2DM patients. We hypothesized that antibiotic periodontal treatments would improve the MCI risk blood sample biomarkers, aside from improving periodontal status and inflammation, in diabetic patients.

The study found an improvement of periodontal parameters in both the intervention and control groups. In the intervention group, IL-6 levels decreased significantly. This result is in line with a previous study that observed a greater significant improvement using antibiotic periodontal treatment compared to scaling-only treatment. 16 17 Another study also highlighted the effect of antibiotic periodontal treatment on diabetic patients to systemic inflammation. 18 Simultaneously with the decrease in IL-6 as a marker of systemic inflammation, several changes in MCI risk blood-based biomarkers were observed. In this study, nine plasma proteins involved in the neurodegeneration process were used to analyze the risk of MCI. ApoA1, TTR, and C3 are plasma proteins involved in the amyloid-beta sequestration process. 11 Meanwhile, ApoC1, A1BG, HPX, A2AP, A2M, and albumin are plasma proteins with functions that involve clearance of brain waste, protection against neurotoxicity, and maintenance of healthy brain vessels. 12 C3 and A2AP levels decreased significantly in the intervention group. In addition, although there was no significant decrease in within-group analysis, there were significant differences in ApoC1, A1BG, and ApoA1 levels between the control group and the intervention group at week 24.

MCI is a transitional stage between the normal cognitive decline of healthy aging and dementia. 4 A significant predictor of MCI is the accumulation of Aβ, which appears decades before dementia caused by AD. 19 20 Both periodontitis and DM are modifiable risk factors for neurodegeneration. They are linked through the systemic inflammation pathway. 21 Through a stronger reaction to bacteremia and dysregulation of the immune inflammatory process in the aging population, DM may enhance the inflammatory response to *Porphyromonas gingivalis* systemically. Given that periodontal infections and their byproducts have been shown to pass the blood–brain barrier and activate neuroglia, these events may also be directly linked to the pathogenesis of neurodegeneration. 22

Plasma levels of TTR and ApoA1 were found to gradually descend the progression of cognitive decline, while C3 levels were observed to decrease during the early stages of cognitive decline. 11 ApoA1 is the primary protein in high-density lipoproteins and is widely known for playing a significant part in reverse cholesterol transport. 23 It is involved in the clearance of Aβ and binds to stop the aggregation and to reduce its toxicity. 24 25 Some studies showed that higher ApoA1 level was found in patients with higher Mini-Mental State Examination (MMSE) scores or good cognitive function. 26 27 However, this study shows opposite results to previous studies. Although there was no significant difference between baseline and week 24, ApoA1 and C3 levels in both the control and intervention groups showed a tendency to decrease. Meanwhile, TTR levels in both the control and intervention groups tended to increase within baseline to week 24, especially in the control group, a significant difference was observed.

In contrast to the changes that occurred in Amyloid-β sequester protein, plasma protein levels related to coagulation (HPX), innate immunity (A1BG), and lipid metabolism (ApoC1) tended to decrease in the intervention group. Meanwhile, these protein levels tended to increase in the control group. In previous research, it was found that elevated levels of these proteins were risk factors for cognitive dysfunction. 28 29 30 31 32 33 However, inconsistent changes occurred in the level of A2AP and A2M in the intervention group.

There are limitations of this study that might influence the result of this study, such as the short period of evaluation time (24 weeks) for cognitive decline. From the previous study, cognitive function changes usually ranged from 3 to 7 years prior to cognitive decline diagnosis, therefore, the fluctuation of blood-based MCI risk biomarkers was observed. 34 35 Although some study observed that the periodontal clinical parameters, including probing depth, clinical attachment loss, and alveolar bone loss, exhibited a positive correlation with cognitive impairment, the direct cause–effect relationship remains unclear. 36 Therefore, it is difficult to determine the direct effect of locally administered antibiotic minocycline on MCI risk yet.

At baseline, the PISA score between the control and intervention groups showed a significant difference. This might be due to the random variations and patients' recruitment challenges. The difference at baseline may have made it difficult to detect the true effect of the intervention and a potential bias.

Other limitations are the limited number of samples, no standardized cognitive decline examination, such as MMSE, was carried out, and the significant differences in PISA score at baseline between the intervention and control groups. In the present study, the cognitive status of each patient is unknown, it is difficult to differentiate between cognitive status. These factors may indicate imbalances in important variables that could confound the result of the study, making it difficult to attribute any observed differences solely to the intervention. However, this study may have a potential impact for older adult's preventive care recommendations. Therefore, further study to investigate the relationship between periodontitis, DM, and cognitive impairment is needed.

## Conclusion

The results demonstrated a significant improvement in periodontal status after the administration of local antibiotic periodontal treatment; however, the changes and improvement in MCI risk-related proteins plasma remained unclear.
